# Fibromuscular Dysplasia/Carotid Web in Angio-CT Imaging: A Rare Cause of Ischemic Stroke

**DOI:** 10.3390/medicina57101112

**Published:** 2021-10-15

**Authors:** Michalina Rzepka, Tomasz Chmiela, Joanna Bosowska, Maciej Cebula, Ewa Krzystanek

**Affiliations:** 1 Department of Neurology, Faculty of Medical Sciences in Katowice, Medical University of Silesia, 40-752 Katowice, Poland; tchmiela@sum.edu.pl (T.C.); ekrzystanek@sum.edu.pl (E.K.); 2 Department of Radiodiagnostics, Invasive Radiology and Nuclear Medicine, Department of Radiology and Nuclear Medicine, School of Medicine in Katowice, Medical University of Silesia, 40-752 Katowice, Poland; jbosowska@sum.edu.pl (J.B.); mcebula@sum.edu.pl (M.C.)

**Keywords:** CT angiography, carotid web, ischemic stroke, angiography, fibromuscular dysplasia

## Abstract

*Background and Objectives*: Carotid web (CaW) is an intimal variant of fibromuscular dysplasia and may constitute as one of rare causes of acute ischemic stroke (AIS). The objective of this study was to determine the prevalence of CaW in patients with AIS or transient ischemic attack (TIA) based on head/neck CT angiography (CTA) in a Polish cohort study. *Materials and Methods*: A retrospective study was performed by analyzing 1480 electronic clinical and imaging data regarding patients with AIS or TIA, hospitalized in the years 2018–2020 in the authors’ institution. The final sample consisted of 181 patients who underwent head/neck CTA; aged 67.81 ± 13.51 years (52% were women). All head/neck CTA studies were independently evaluated by two radiologists. The patient’s clinical condition was assessed with the National Institutes of Health Stroke Scale (NIHSS, 5.76 ± 4.05 and 2.88 ± 3.38 at admission and at discharge, respectively). *Results*: 27 patients were identified with CaW. The prevalence of CaW in the final sample (181 pts with good quality CTA) was 14.9%. In the CaW group, 89% patients had AIS, including 26% diagnosed with recurrent and 11% with cryptogenic strokes. There were no significant differences between the presence of CaW and gender, age, NIHSS score, recurrent or cryptogenic stroke. *Conclusions:* Our study demonstrated that CaW may be an underrecognized entity leading to cerebrovascular events. The diagnosis of CaW depends on a high level of awareness and a comprehensive analysis of the neuroimaging studies. Our findings support the hypothesis that it is worthwhile to perform CTA to determine the etiology of ischemic stroke, particularly if predicting factors were not identified.

## 1. Introduction

Cerebrovascular diseases are one of the major public health problems in industrialized countries. Stroke is a leading cause of permanent disability among adults and the third leading cause of death in Poland, affecting nearly 80,000 individuals annually [1,2]. Acute ischemic stroke (AIS) is defined as a sudden onset of neurologic deficit caused by focal brain ischemia with imaging evidence of acute infarction lasting more than 24 h (unless interrupted by death or treatment) [3]. 

An ischemic episode with neurologic deficits but without acute infarction defines transient ischemic attack (TIA) [3]. Up to 80% of strokes are attributed to brain ischemia, caused by the pathology of large vessel atherosclerosis, small vessel disease, cardiovascular embolism or other rare causes. About 30% cases of strokes are cryptogenic, in which the cause cannot be evidently established [4].

One rare vessel disease that can be recognized as a cause of stroke is fibromuscular dysplasia (FMD). It is an idiopathic, noninflammatory, nonatherosclerotic vascular disease [5]. FMD most commonly involves the renal, visceral, and craniocervical arteries. It occurs generally in females, middle-aged, and Caucasians [6]. The prevalence of FMD is not well-established in the general population. A study reported that the incidence of carotid FMD ranged from 0.9% to 5.6% in a population of patients with carotid artery dissection [7]. The most common clinical manifestation of FMD is renovascular hypertension and less frequently neurological symptoms, such as headaches, dizziness, TIA or AIS. FMD can be asymptomatic as well [5].

Carotid web (CaW) is a rare subtype of unifocal FMD and remains as an under-recognized cause of cryptogenic and recurrent ischemic stroke in younger patients without any risk factors [8]. CaW, originally described in 1964 by Palubinskas and Ripley, is defined as a shelf-like defect in a linear filler at the posterior part of the internal carotid artery (ICA) on catheter angiography [9]. Digital subtraction angiography (DSA) is a gold standard for diagnosis of FMD, but current diagnostics generally rely on computed tomography angiography (CTA) [10].

Thus far, it appears that the occurrence of FMD in the carotid arteries has been underestimated in the population of Polish patients with AIS or TIA. In this perspective, we aim to assess the prevalence of CaW among patients with AIS or TIA based on the head and neck CTA and to characterize the clinical features of patients with CaW.

## 2. Materials and Methods

We performed a retrospective analysis of all patients with AIS or TIA admitted to Central Clinical Hospital of the Medical University of Silesia in Katowice from January 2018 to December 2020. The initial group consisted of 1480 individuals. In the first stage, 1092 patients (pts) without both head and neck CTA were excluded. Then, in a 380 pts study group, 199 pts met the exclusion criteria, which included the conditions preventing a reliable assessment, such as massive atherosclerotic plaques (*n* = 92), lack of carotid CTA (*n* = 63), occlusion of common carotid artery (CCA) and/or ICA (*n* = 30), movement artifacts (*n* = 9) and ICA stent (*n* = 5). 

In the final group of 181 pts, clinical data regarding gender, age, National Institutes of Health Stroke Scale (NIHSS) score [11], Modified Rankin Scale (mRS) score [12], the stroke side, treatment, bloodwork results and risk factors were collected. Those pts underwent carotid Doppler ultrasound examination. Patients were evaluated for cryptogenic stroke in accordance to criteria of Trial of ORG 10172 in Acute Stroke Treatment (TOAST) [4].

The next step was composed of a blinded reevaluation of patient carotid CTA examinations to find CaW. As there are no formal radiological diagnostic criteria, “string-of-beads”, “web-like” defects and focal/tubular lesions other than plaques were classified as CaW [13]. It was performed by two radiologists with 7 and 3 years’ experience in CTA evaluation on licensed diagnostic workstations (Advantage Workstation (AW) 4.4 Software, GE Healthcare, Waukesha, WI, USA). Exclusion criteria included conditions preventing a reliable assessment, such as massive atherosclerotic plaques (*n* = 92), lack of carotid CTA (*n* = 63), occlusion of common carotid artery (CCA) and/or ICA (*n* = 30), movement artifacts (*n* = 9) and ICA stent (*n* = 5).

**Figure 1:**
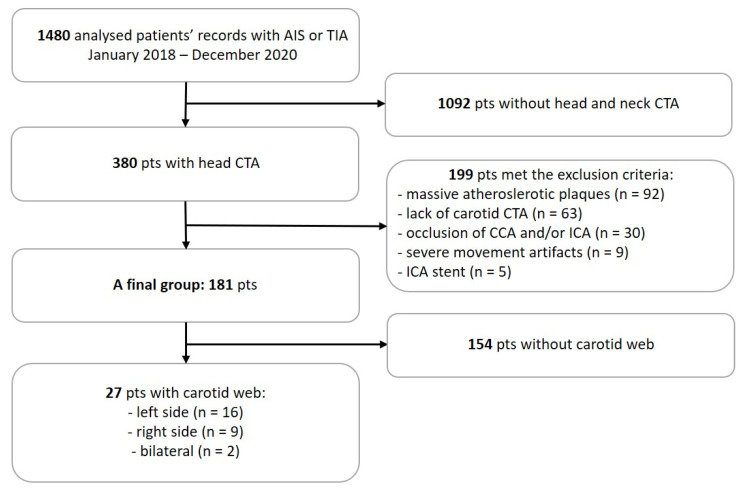
A final group creation process to detect carotid web in patients with AIS or TIA. AIS—acute ischemic stroke; TIA—transient ischemic attack; pts—patients; CTA—computed tomography angiography; CCA—common carotid artery; and ICA—internal carotid artery.

The study group consisted of 181 patients; 94 females (52%) and 87 males (48%), aged 67.81 ± 13.51 years (71.99 ± 13.30 years and 63.30 ± 12.29 years, respectively) (mean ± SD). The process of the final group creation is presented in Figure 1.

The statistical analysis was performed with Statistica 13.3 (TIBCO Software Inc. (2017) Statistica (data analysis software system, version 13. http://statistica.io)). The quantitative variables are presented as an arithmetic mean and a standard deviation (normally distributed variable) or a median and the interquartile range (variables of not normal/skewed distribution). The normality of distribution was assessed with the Shapiro–Wilk test. Qualitative variables are presented as absolute values and percentage. 

The intergroup differences for the quantitative variable were assessed with an analysis of variance (normally distributed variables) or the U-Mann–Whitney or the Kruskal–Wallis test (variables of skewed distribution). In the case of statistically significant differences within many groups revealed by the Kruskal–Wallis test, a post-hoc type analysis was performed. Fisher’s exact test or chi-square test were performed for qualitative variables. Relationships of quantitative variables were assessed with the Spearman’s rank correlation coefficient. Statistical significance was established at *p* < 0.05.

Due to the retrospective character of the work and data anonymization, the Ethics Committee of Medical University of Silesia waived the requirement to obtain ethical approval for this study.

## 3. Results

### 3.1. The Prevalence of CaW

In the study group, we found 27 CaWs incuding 16/27 CaWs on the left side and 9/27 CaWs on the right side; 2/27 CaWs were bilateral. The prevalence of CaW was 1.8% in the overall examined cohort, whereas, in the study group, in which we were able to detect CaW, the prevalence of CaW was 14.9%.

In the CaW group 89% patients were diagnosed with AIS. We found 10/20 of strokes on the left side, 10/20 were on the right side, and 5/20 patients had CaW on the same side as the cerebral infarction. The CaW side was close to significance with the side of the ischemic stroke (*p* = 0.058); however, due to the low number of cases in each of the analyzed subgroups, this should not be considered credible. We found that 25% (5/20) stroke patients had CaW on the ipsilateral side as the cerebral infarction. Among them, three patients had cryptogenic stroke, which may indicate a potential etiology of CaW as a risk factor. The complete characteristic of final study group is shown in Table 1.

Males were significant younger than females and had better outcomes (lower mRS score). Atrial fibrillation was more frequent in women than in men. (Table 2.)

### 3.2. CaW and Non-CaW Groups

The occurrence of CaW was associated with a higher level of total cholesterol and high-density lipoprotein cholesterol. There were no significant differences between patients with and without CaW as we considered gender, age, NIHSS score at admission and at discharge or mRS score. We found no significant differences between patients treated with recombinant tissue plasminogen activator (rtPA) or thrombectomy.

### 3.3. CaW and Cryptogenic and Recurrent Stroke

In the CaW group, there were 7/27 (26%) recurrent strokes and 3/27 (11%) cryptogenic strokes. No significant differences in CaW patients with cryptogenic or recurrent strokes were observed.

### 3.4. CaW and Risk Factors

There were no significant differences in cardiovascular risk factors, such as hypertension, dyslipidemia, atrial fibrillation, diabetes mellitus, previous myocardial infarction, ischemic heart disease, smoking and arterial narrowing between the groups with CaW and without CaW.

### 3.5. AIS and TIA Groups

The analysis of AIS and TIA groups showed no significant differences in the occurrence of CaW.

## 4. Discussion

To our knowledge, this is the first study to extensively assess the occurrence of CaW in cohort of Polish patients with acute cerebrovascular events (AIS/TIA), retrospectively. Our study showed that the diagnosis of CaW was highly underestimated as we considered the causes of ischemic strokes. It corresponds to Wojcik’s reports, which clearly show that familiarity with this clinical problem varies significantly between different specializations; however, generally, the knowledge about this pathology is low [14].

Our study suggests that CTA were not evaluated in detail for the occurrence of CaW routinely at admission as CaWs are usually not associated with significant stenosis [8]. This result highlights that little is known about the CaW/FMD as a rare cause of ischemic stroke. By the reevaluation of CTA, we found 27 patients with CaW. Our retrospective study showed the prevalence of CaW was 1.8% in the overall cohort (all admitted patients with acute cerebrovascular events), although not every patient had CTA performed. 

This result is in the line with other studies of Mei et al., which reported the prevalence of CaW of 1.6% in US cohort of patients with ischemic stroke (IS) [15] and 2.2% in Asian cohort with IS [16]. However, our final study group, which consisted of the patients who met the rigorous inclusion criteria, was much numerous (14.9%). Likely, such high results were achieved because the CTA scans were retrospectively reevaluated by radiologists.

CaW appears to have diagnostic challenges because it may not be detected by routine neuroimaging techniques. In recent years, imaging methods have developed, and now we can diagnose craniocervical vascular pathology with a variety of imaging methods. Catheter digital subtraction angiography (DSA) remains the gold standard method to detect and accurately assess CaW due to the best spatial and contrast resolution; however, because of its invasiveness and high exposure to ionizing radiation, it is less useful in daily routines [17]. Moreover, the range of interest (ROI) during the DSA study is limited to intracranial arteries in order to have as little impact on a person as possible/radiological protection of the patient. Due to the fact that bifurcation of the common carotid artery is often outside a scan area, we cannot assess CaW in routinely performed DSA.

It seems reasonable to use this invasive imaging method for final verification in uncertain cases. As we consider non-invasive methods, our patients had ultrasound (US) Doppler examination of carotid arteries, and we found out patients that were detected with CaW in this study. US is a low-sensitivity method, strongly dependent on the skills and experience of the operator. Therefore, it is not widely used in clinical practice to detect CaW [14]; however, in selected cases, it may be a sufficient method [18]. 

The most useful method in the diagnosis of CaW in daily practice appears to be CT angiography (CTA) [8]. The examination is well tolerated by patients, widely available, associated with a low risk of complications and is routinely performed in the acute phase of ischemic stroke [19,20]. According to some reports, its sensitivity does not differ significantly from that of the DSA examination [21]. CTA should be considered as the first-choice examination, and its current widespread use is confirmed by Wojcik’s reports [14].

Our study found no statistically significant differences in demographics, stroke severity, or vascular risk factors between the groups with CaW and without CaW. Mei et al. also did not find any differences regarding atrial fibrillation and coronary heart disease between those patients [15]. The higher levels of LDL cholesterol and total cholesterol in the patients with CaW seem to be clinically insignificant. 

Other studies confirmed that TIA/AIS can be more common in CaW patients, even with fewer risk factors [22,23]. However, usually the number of patients with CaW was small, so it was very difficult to determine the predicting factors [23,24]. CaWs pose a serious risk of ischemic stroke, probably due to thrombus development associated with turbulences with the blood flow and stagnation [8]. The optimal management strategy to prevent AIS in patients with CaW is still under investigation. Antiplatelet therapy, carotid angioplasty, invasive treatment (carotidangioplasty, stenting, mechanical thrombectomy or endarterectomy) are the therapeutic options that can be considered. In our study, an antiplatelet therapy (monotherapy) was administered in the majority of patients as the secondary stroke prevention. 

Despite this, some patients had a recurrent stroke, which demonstrates that it could be an insufficient prevention. Studies comparing different pharmacotherapy regimens are needed to determine the optimal pharmacological prevention. However, surgical intervention is supposed to be a dedicated method of treatment of CaW [25]. Haussen et al. reported the recurrence rate of 29% in patients treated with pharmacotherapy, whereas no recurrent stroke/TIA was observed in stented patients [22]. If CaW is found, looking for FMD in other locations should be considered [26].

Our study has limitations. First of all, the diagnosis of CaW in our final group was based on CTA without DSA or histopathological confirmation. Secondly, because of the retrospective nature of single-center study, selection and sampling bias was possible. The final study group accounted for 12.23% of the entire group (*n* = 181/1480). It is notable that our finding can be underestimated due to the fact that CTA was not performed in the majority of patients with AIS/TIA. In the final group of patients with CTA, the occurrence of CaW may be underestimated due to exclusion criteria.

## 5. Conclusions

Our study demonstrated that CaW may be an underrecognized entity leading to cerebrovascular events. The diagnosis of CaW depends on a high level of awareness and a comprehensive analysis of the neuroimaging studies. Our findings support the hypothesis that it worthwhile to perform CTA to find the etiology of ischemic stroke particularly if predicting factors were not identified.

## Figures and Tables

**Table 1:** Demographic, clinical and laboratory characteristic of a final analyzed post-stroke and TIA group.

	Study Group (*n* = 181)		
Variable	CaW (*n* = 27)	no-CaW (*n* = 154)	*p* Value
Gender [*n* (%)]			0.39
Females	16 (59%)	78 (51%)	
Males	11 (41%)	76 (49%)	
Age [years] [*n* ± SD]	66.70 ± 14.34	68.01 ± 13.40	0.73
Pathology [*n* (%)]			
Transient ischemic attack	3 (11%)	17 (11%)	-
Acute supratentorial ischemic stroke	20 (74%)	122 (79%)	-
Acute infratentorial ischemic stroke	4 (15%)	15 (10%)	-
NIHSS—admission [*n* ± SD]	5.76 ± 4.05	7.51 ± 5.83	0.27
NIHSS—discharge [*n* ± SD]	2.88 ± 3.38	5.14 ± 6.14	0.13
rtPA [*n* (%)]	17 (63%)	74 (48%)	0.12
Thrombectomy [*n* (%)]	3 (11%)	19 (12%)	0.94
Stroke side (supratentorial) [*n* (%)] ^1,2^			0.89
Right	10 (37%)	59 (38%)	
Left	10 (37%)	63 (41%)	
Recurrent stroke [*n* (%)]	7 (26%)	28 (18%)	0.38
Cryptogenic stroke [*n* (%)]	3 (11%)	20 (13%)	0.99
Risk factors [*n* (%)]			
Hypertension	23 (85%)	129 (84%)	0.97
Dyslipidemia	14 (52%)	80 (52%)	0.71
Atrial fibrillation	5 (19%)	25 (16%)	0.64
Diabetes mellitus	4 (15%)	48 (31%)	0.12
Smoking	5 (19%)	33 (21%)	0.87
Myocardial infarction	3 (11%)	27 (18%)	0.49
Ischemic heart disease	8 (30%)	62 (40%)	0.43
Arterial narrowing	8 (30%)	32 (21%)	0.22
Bloodwork results [*n* ± SD]			
LDL cholesterol [mg/dL]	130.08 ± 46.49	112.97 ± 49.38	0.06
HDL cholesterol [mg/dL]	52.96 ± 12.43	47.64 ± 15.60	0.02
Total cholesterol [mg/dL]	209.04 ± 50.33	183.65 ± 57.36	0.02
Triglycerides [mg/dL]	125.59 ± 65.05	124.88 ± 59.53	0.65
Hb A1c [%]	6.50 ± 0.43	7.27 ± 1.99	-

SD—Standard Deviation, TIA—Transient Ischemic Stroke, NIHSS—National Institutes of Health Stroke Scale, rtPA—recombinant tissue plasminogen activator, LDL—Low-density lipoprotein, HDL—High-density lipoprotein, HbA1c—hemoglobin A1c, ^1^—two cases of bilateral CaW were not included in the table, and ^2^—19 cases of infratentorial stroke were not included in the Table. U-Mann–Whitney test for quantitative variables and Fisher’s exact test for qualitative variables.

**Table 2:** Baseline characteristics of men and women in the final group of patients with AIS/TIA.

	Study Final Group (*n* = 181)		
Variable	Females *n* = 94 (52%)	Males *n* = 87 (48%)	*p* * Value
Age [years] [*n* ± SD]	72.00 ± 13.30	63.30 ± 12.29	<0.01
Pathology [*n* (%)]			
Transient Ischemic Attack	12 (13%)	8 (9%)	0.24
Acute supratentorial ischemic stroke	74 (79%)	68 (78%)	0.96
Acute infratentorial ischemic stroke	8 (8%)	11 (13%)	0.39
NIHSS—admission [*n* ± SD]	8.04 ± 6.32	6.38 ± 4.64	0.09
NIHSS—discharge [*n* ± SD]	5.77 ± 6.75	3.77 ± 4.54	0.09
mRS [*n* ± SD]	2.59 ± 2.05	1.86 ± 1.80	0.02
rtPA [*n* (%)]	48 (51%)	43 (49%)	0.54
Thrombectomy [*n* (%)]	12 (13%)	10 (12%)	0.69
Supratentorial stroke site [*n* (%)]			0.10
Right	31 (33%)	38 (44%)	
Left	43 (46%)	30 (35%)	
Recurrent stroke [*n* (%)]	18 (19%)	17 (20%)	0.93
Cryptogenic stroke [*n* (%)]	9 (10%)	14 (16%)	0.23
Risk factors [*n* (%)]			
Hypertension	79 (84%)	73 (84%)	0.98
Dyslipidemia	49 (52%)	45 (52%)	0.96
Atrial fibrillation	24 (26%)	6 (7%)	<0.01 *
Diabetes mellitus	26 (28%)	26 (30%)	0.74
Smoking	14 (15%)	24 (28%)	0.04
Myocardial infarction	13 (14%)	17 (20%)	0.30
Ischemic heart disease	38 (40%)	32 (37%)	0.62
Arterial narrowing	17 (18%)	23 (26%)	0.18
Bloodwork results [*n* ± SD]			
LDL cholesterol [mg/dL]	120.09 ± 51.67	110.86 ± 46.20	0.29
HDL cholesterol [mg/dL]	51.15 ± 12.30	45.51 ± 17.52	<0.01
Total cholesterol [mg/dL]	196.08 ± 58.36	178.46 ± 54.17	0.05
Triglycerides [mg/dL]	120.28 ± 59.71	130.17 ± 60.75	0.26
Hb A1c [%]	7.40 ± 2.05	6.6 ± 1.14	-

SD—Standard Deviation, AIS—Acute Ischemic Stroke, TIA—Transient Ischemic Attack, NIHSS—National Institutes of Health Stroke Scale, mRS—modified Rankin Scale, rtPA—recombinant tissue plasminogen activator, LDL—Low-density lipoprotein, HDL—High-density lipoprotein, HbA1c—hemoglobin A1c, and *—result with lower reliability due to few number of cases in the subgroup. U-Mann–Whitney test for quantitative variables and Fisher’s exact test for qualitative variables.

## Data Availability

The data presented in this study are available on request from the corresponding author.
